# Real-World Utilization of the HCV Care Cascade Before and After Implementation of a Program to Streamline Care and Promote Treatment

**DOI:** 10.3390/v18050499

**Published:** 2026-04-24

**Authors:** Daniella Rahamim-Cohen, Ayelet Aviva Basson, Clara Weil, Izana Kaplan-Lavi, Odelia Tassa-Liani, Yael Topol, Gabriel Chodick, Bar Cohen, Limor Adler, Shirley Shapiro Ben David

**Affiliations:** 1 Medical Division, Maccabi Healthcare Services, Tel Aviv 6812509, Israelshirley.shap@gmail.com (S.S.B.D.); 2 Kahn Sagol Maccabi Research and Innovation Center, Maccabi Healthcare Services, Tel Aviv 6812509, Israel; 3 Gilead Sciences Israel Ltd., Hod Hasharon 4524075, Israel; 4 Gray Faculty of Medical and Health Sciences, Department of Family Medicine, Tel Aviv University, Tel Aviv 6997801, Israel

**Keywords:** hepatitis C, care cascade, DAA treatment, HCV

## Abstract

Objectives: The World Health Organization (WHO) goal of eradicating hepatitis C virus (HCV) infection by 2030 has encouraged healthcare providers to implement proactive strategies to improve diagnosis and treatment. The aims of this retrospective cohort study were to assess a program designed to improve the HCV care cascade and facilitate access to treatment, within a national healthcare provider in Israel, Maccabi Healthcare Services (MHS). Methods: Included were adult patients newly diagnosed with HCV infection before and after the implementation of a screening and care optimization program. Patients diagnosed in 2017 served as the reference group (RG), while those diagnosed in 2019 (following the program implementation) comprised the intervention group (IG). Study outcomes included completion of HCV laboratory testing, time to consultation with gastroenterologist/hepatologist (GE), and initiation of treatment with direct-acting antivirals (DAAs). Results: The study sample included 356 HCV Ab+ patients in the RG (median age = 46 years; 41% females), and 328 in the IG (median age = 48 years; 39% females). Compared to RG, IG demonstrated higher rates of patient visiting GE visit (78.1% vs. 63%) and initiating DAA treatment (66.3% vs. 35.5%). Conclusions: Implementation of a restructured HCV care cascade was associated with a greater proportion of patients receiving expert consultation and higher DAA treatment uptake, important steps towards HCV eradication.

## 1. Introduction

Hepatitis C is one of the major viral infections affecting quality of life and life expectancy globally. The primary method of transmission for the virus is exposure to bodily fluids, with the most common routes being infected needles and medical equipment, or blood transfusions. There are an estimated 50 million people worldwide who have chronic hepatitis C, with approximately 1 million new cases yearly, and over 200,000 deaths annually [1]. Presentation can be acute or chronic and varies in its severity. Severe chronic cases can result in cirrhosis, liver decompensation and hepatocellular carcinoma. These features and the public economic burden they cause make HCV one of the most pressing public health concerns, globally.

Up until 2015, the main treatment for HCV infection included interferon for 6–12 months, with a limited eradication success and potentially serious side effects [2]. The development of direct-acting antiviral (DAA) agents, enabling actual cure following a short oral treatment course [3,4], was revolutionary, with relatively tolerable side effects. For the first time ever, the notion that HCV can be eradicated became realistic.

Subsequently, in 2016, the WHO stated its goal of eliminating HCV infection by 90% by 2030 [5]. Many countries, including Israel, aligned with this goal and began planning and implementing strategies to accomplish it [6]. Initial guidelines for HCV eradication were published by the WHO in 2016, and those were later updated in 2018 with the introduction of pan genotypic DAAs [7], recommending their use in patients with chronic HCV infection aged 18 or older.

As it is now possible to cure HCV, and thereby halt the spread of the virus thanks to DAAs, the main barriers for HCV cure are now mainly logistic and delivery issues [8,9]. Identified barriers can be at the systemic level, provider level and patient level, and accordingly, various processes can be designed to overcome them [10]. As the diagnosis and treatment for HCV includes multiples steps and various types of healthcare resources, care cascades have been focused on improving the delivery and availability of HCV care [11]. Several countries have implemented programs directed at their specific risk populations, healthcare services construct, and other considerations with varying degrees of success [12,13]. In the United States, only about a third of insured patients diagnosed ultimately get treated [14], prompting the Biden proposal for a new 5-year program to eliminate HCV [15]. Egypt, on the other hand, was the first country to achieve the WHO elimination goal [16], illustrating the global variability in conceptualizing and implementing screening, diagnosis and treatment plans.

In accordance with the abovementioned developments, in 2019, Maccabi Healthcare Services (MHS), the second largest state-mandated care provider in Israel with over 2.6 million members nationwide, designed and implemented a program to identify and treat eligible HCV positive patients. All residents in Israel are entitled to cover under the healthcare basket and are members of one of four care providers, with each MHS member having a named family physician. This structure enables easier patient outreach and long term follow-up. Uniquely, the main risk group in the country is recognized to be former Soviet-bloc immigrants, rather than the usually recognized risk groups including IV drug users, prisoners or men who have sex with men [17,18]. The MHS community-based program focused primarily on streamlining diagnosis, care and treatment processes. The aims of this retrospective cohort study were to assess MHS’s program, designed to improve the HCV care cascade and facilitate access to treatment.

## 2. Methods and Materials

### 2.1. The New HCV Care-Cascade in MHS

**Figure 1:**
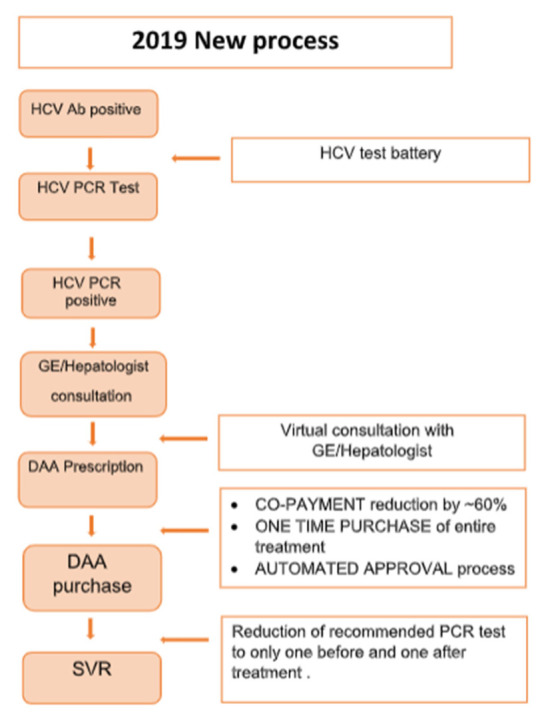
Diagram depicting the new process implemented in 2019.

The new care cascade was initially designed and conceptualized by systematically reviewing previous treatment programs and processes, identifying barriers and removing them where possible (see Figure 1).

1. Centralized management: A task force at MHS developed and oversaw HCV diagnosis and care processes in accordance with the WHO guidelines and goals, and the national HCV elimination program as guided by the Israeli Ministry of Health. Each of MHS’ districts were asked to form a dedicated team for HCV diagnosis and care program, and regular meetings were held.
2. Test requests: A specific HCV test battery, including relevant tests (CBC, LFTs, HCV- PCR) was introduced into the EMR (electronic medical record) to simplify requesting them by the physician, following an initial positive antibody test.
3. Virtual Gastro/hepatology consultations: We added a virtual gastroenterology/hepatology consultation in April 2019, whereby the family physician can send a query to the specialist via the EMR and receive a reply within 72 h. This helps bypass long waiting time and insufficient access to specialist care for certain populations. The specialist would recommend the medication, and it would then be approved for dispensation of the medication with a family physician prescription.
4. Automated medication approval: Once a hepatology consultation was done, the medication approval process would be submitted into the EMR, and approval was usually given within a couple of days.
5. Payments: In mid-2019 and in light of cost being a potential barrier to care (based on reports of clinicians and staff), MHS decided to reduce co-payment costs by 60% and pre-approving the entire course of medication from the start, rather than requiring a monthly pharmacy visit; thus reducing logistic and financial barriers to care. This also resulted in patients having to pay only one co-payment rather than potentially two (medication co-payments have a cap per quarter, and obtaining medication over two quarters often resulted in two co-payments).
6. PCR testing: We removed the need for serial PCR testing during treatment as per guidelines.
7. External collaborations: We established communication and work processes with the Ministry of Health, prison authorities, rehab clinics, and liver patient advocacy groups, in order to widen our reach and maximize our ability to access MHS patients who might be at risk for HCV. Collaboration focused on identifying potential patients, and tailoring ways to ensure they go through cascade steps as easily as possible.
8. Oversight: The teams regularly and proactively reviewed all PCR positive patients and followed up on referrals and purchases, calling patients and physicians to make sure that patients were compliant and no logistic barriers were stopping them from receiving care. In short, the care cascade was constantly reviewed and whenever things were not going smoothly, immediate action was taken to mend it. Regular follow-up meetings were conducted to monitor progress within the care cascade, making the patient a likewise active player in his care.

### 2.2. Participants

Using the MHS database, we identified all adult patients newly diagnosed with positive HCV antibodies before and after the implementation of a screening and care optimization program that started on 1 January 2019. Patients diagnosed in 2017 served as the reference group (RG), while those diagnosed in 2019 comprised the intervention group (IG). The study was approved by the Maccabi Healthcare Services Helsinki committee, MHS-0054-21. Informed consent was waived by the committee. Excluded were all patients listed as having active malignant disease according to the MHS registry or were hospitalized for ≥30 days during the follow-up period. All patients were MHS members for one year before and one year after their first positive HCV antibody test.

### 2.3. Medication Eligibility

The prescribing of DAAs was approved and partially subsidized under the healthcare basket in Israel from 2015 onwards in stages depending on liver fibrosis stage and virus genotype. Meaning, eligibility for subsidized care gradually increased throughout our study period. Most patients were included in the eligibility criteria by 2017, with the last groups being covered by the end of the year. In 2018, all patients with a positive HCV PCR result were eligible for subsidized treatment with DAAs. As our follow-up period was one year after diagnosis, all study participants were eligible during this period

### 2.4. Study Variables

The following steps in the HCV care cascade were defined: diagnosis (positive HCV antibody tests, positive HCV PCR test), designing a care plan (gastroenterologist/hepatologist visit, either in-person or virtual), initiation of treatment (DAA prescription, DAA purchase), and confirmation of treatment success (SVR test, defined as a negative HCV PCR test 8–52 weeks after the end of treatment). The 8–52 week time frame was chosen to enable a longer time frame for SVR measurement. The number of patients progressing through these steps within a year from receiving a positive HCV antibody test was compared between two cohorts. Patient demographics included age, sex, residential socioeconomic status (SES), district of residence, birth region, and smoking status. Socioeconomic status was determined based on patient addresses and categorized according to the definitions set by the Central Bureau of Statistics [17].

Additional clinical characteristics recorded any time before the first positive HCV antibody test included diagnoses of hepatitis A and B, human immunodeficiency virus (HIV), cardiovascular disease (CVD), chronic kidney disease (CKD), diabetes, chronic obstructive pulmonary disease (COPD), hypertension congestive heart failure (CHF), human papillomavirus (HPV), chlamydia, syphilis, gonorrhea, and genital herpes. These were collected due to the epidemiological risk factors and common infection mechanisms. Patients who received treatment without a recorded positive PCR test were added to the total number of patients treated, as DAAs cannot be dispensed without a valid PCR test. We surmise that their test was taken in the hospital or prison setting and sent to MHS manually rather than via the electronic medical record.

### 2.5. Statistical Analysis

We report descriptive statistics in frequencies (“*n*”, %), mean (SMD), or median (IQR), where relevant according to the type of variable for all patient demographic and clinical variables at the time of the first positive HCV antibody test. Comparison of RG and IG were done using Fisher Exact test and 95% confidence interval.

All analyses were conducted using IBM-SPSS version 25.

## 3. Results

### 3.1. Descriptive Statistics

A total of 356 eligible patients were included in the RG (median age = 46 (35–55) years; 41% females), and 328 in the IG (median age = 48 (39–57) years; 39% females). A positive HCV PCR test was documented in 56.2% and 48.8% of RG and IG cohorts, respectively. The study cohorts were comparable in smoking rates and demographic characteristics. In addition, various chronic and infectious medical conditions were compared between the groups, with no significant difference found between the two groups (the full data is presented in Table 1).

### 3.2. Progression in the Care Cascade Within 12 Months

**Figure 2:**
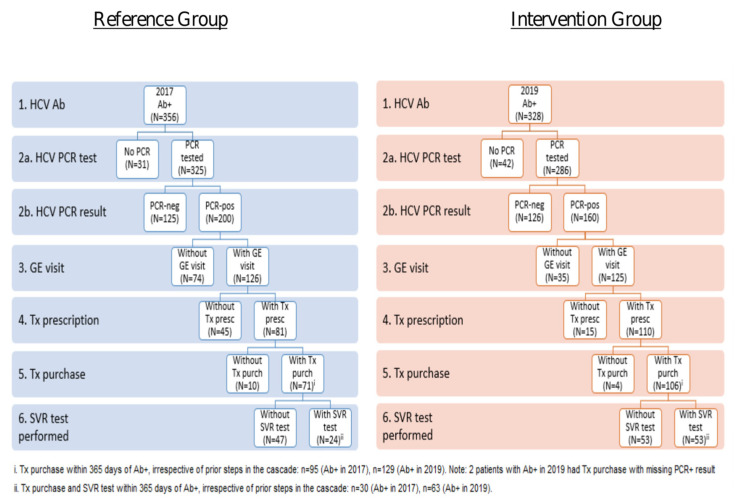
Progression through the care cascade within 12 months of HCV antibody positive result in 2017 (reference group) vs. 2019 (intervention group). CI = confidence interval Tx = treatment, GE = gastroenterology.

**Figure 3:**
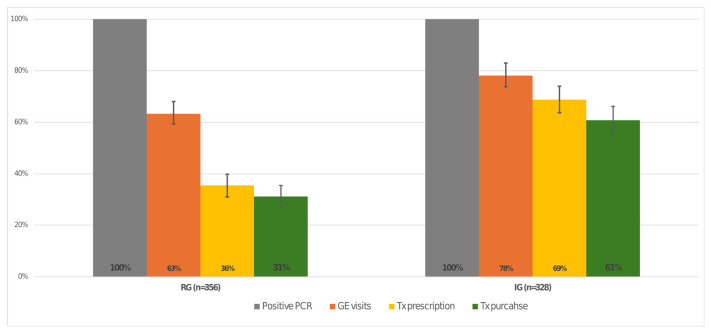
Percentage and 95% CI of patients with positive PCR HCV to progress through the care cascade steps until treatment purchase. CI = confidence interval.

PCR testing was done for 325 patients in the RG cohort, and for 286 patients in the IG cohort (91.5% compared to 87.2% respectively). Within the RG participants tested, 61.5% (n = 200) were PCR positive compared to 55.9% in the IG cohort (n = 160). Of the positive HCV antibody and positive HCV PCR patients in the RG, 63% (n = 126) patients visited a gastroenterologist/hepatologist (GE), compared to 78.1% in the IG (n = 125). Of patients with positive antibody and PCR HCV tests seen by the specialist in the RG group, 64.3% (n = 81) were prescribed the appropriate treatment and 87.65% of them fulfilled it (n = 71), compared to 88% (n = 110) of the IG who were given a prescription and 96.7% (n = 106) who fulfilled it. Of patients who purchased treatment, 33.8% (n = 24) had an HCV PCR test to assess SVR (irrespective of the result) in the RG compared to 50% (n = 53) of the IG (Figure 2 and Figure 3). All differences were significant with a *p* value of <0.001.

### 3.3. Treatment Completion Irrespective of Cascade Steps

**Figure 4:**
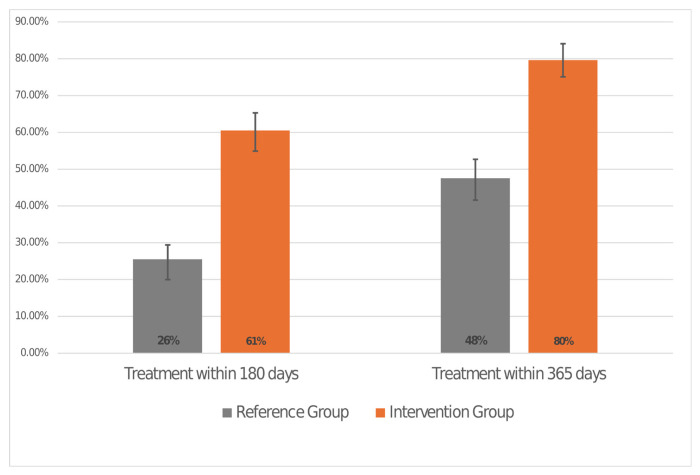
Proportion and 95% CI of patients treated for HCV within 180 days and 360 days for reference group (RG) vs. intervention group (IG). CI = confidence interval.

Among all the patients with positive antibody and PCR tests (including two who were treated without a PCR test), treatment within 365 days of first positive antibody test was recorded among 47.5% (n = 95) RG compared to 79.6% (n = 129) in the IG, irrespective of the intermediate cascade steps (see Figure 4). Differences were significant with a *p* value of <0.001.

## 4. Discussion

HCV infection has long been recognized as a prevalent global public health concern, causing significant morbidity and mortality [2,5,7]. The introduction of pan-genotypic DAA treatment has made eradication of the disease and its outcomes possible, as reflected by the WHO’s ambitious goal of HCV eradication by 2030. In alignment with this objective, MHS implemented an improved care cascade for HCV diagnosis and treatment in 2019. This process was based on previous projects and experience within MHS [18], while also addressing additional potential barriers as described extensively in the literature [9,10,19,20].

The redesigned care cascade targeted barriers at multiple levels: Patient-based barriers were dealt with by reducing co-payments, dispensing the entire medication course, and targeted patient education. Provider-based barriers were mitigated through introducing virtual gastroenterologist consultation, systematized test ordering, automated medication approval, and enhanced clinical oversight. System barriers were dealt with through collaboration with the Ministry of Health, prison healthcare services, and patient advocacy groups from diagnosis to treatment and implementing improvements where possible, in order to streamline the care cascade and ensure linkage to care.

Our results indicate that the new program was related with a 30% improvement in the number of treated patients during the period in which the improved care cascade was implemented as compared to the reference group. The two cohorts were similar in sociodemographic characteristics, with no significant differences observed in the parameters examined, including chronic comorbidities and history of sexually transmitted infections, supporting their comparability. One of the most important sociodemographic characteristics in this case is country of birth; as it has been well-recognized as relevant for HCV prevalence [4]. A study by Batash [21], referring specifically to USSR immigrants in New York, demonstrates an HCV infection prevalence of 28.6%, likely due to insufficient sterilization practices in healthcare. In Israel, approximately 15% of the population is made up of individuals born in the former USSR and HCV prevalence has been shown to be significantly higher in this group [22,23,24]. In this study, 25.6% of RG patients and 28.4% of IG patients were reported to have been born in former USSR countries. As part of the improved care cascade, this factor was taken into account and patients were approached in their native language, when necessary to improve engagement and linkage to care.

The percentage of HCV PCR positive patients among antibody positive patients is typically around 70% [25]. In this study, we report a lower proportion, approximately 52% (56.2% in RG and 48.8% in IG). It was suggested that spontaneous HCV clearance levels of up to 65% can occur if the infection happened before the age of 5 [26]. As many of the patients born in former USSR countries were likely infected in infancy due to contaminated needles used in vaccination practices [21,22,23] this may explain the lower rates of PCR positive results in antibody positive patients.

Among PCR positive patients, a higher percentage of patients in the IG consulted a GE specialist, were prescribed treatment and purchased it compared to the RG. One of the major differences between the two groups is the introduction of the virtual hepatology consultations in April 2019. This enabled family physicians to send a direct query via the EMR to the specialist regarding suggested treatment and receive a reply within 3 days. As waiting times for in person consultations can be up to months, this was likely instrumental in shortening treatment times and improving compliance [27,28], and has been recognized specifically in HCV treatment to increase treatment access [29]. The expedited approval process once medication had been issued most probably further shortened time to treatment dispensation. The higher percentage of patients purchasing medication is likely due to the 60% reduction in co-payment, thus enabling making medication more affordable. Purchasing the entire course from the beginning, as compared to the previous method of monthly dispensation, enabled patients to pay only one co-payment, rather than one with each dispensation, thus improving accessibility and the likelihood of completing the course. The benefits of these changes are also reflected in the higher number of patients to be treated within 180 days.

The study outcomes showed that regardless of whether patients went through every step in the care cascade, more patients were treated in the IG than in the RG, suggesting an additional benefit in improving individual care cascade steps, as multiple studies have shown [30,31]. Furthermore, the IG results for patients treated are substantially higher than the 21–50% range found in multiple reports describing HCV treatment outcomes [20,32,33,34], further suggesting that our new process was effective. Post-treatment SVR testing completion between the two groups is also improved in the IG compared to the RG, although recording SVR was not defined as a specific goal, based on the assumption that 95–99% of patients treated with DAAs reached SVR [35]. The overall low number of patients to undergo SVR testing in both groups may stem from the less stringent follow-up and oversight for the test, as resources were mainly focused on diagnosis and treatment.

The study additionally highlights the significant role family physicians play in the treatment of HCV. By improving the workflow and support for the family physician in diagnosis and treatment, they were more aware and involved in diagnosis and treatment. This was enhanced in the 2019 program by multiple webinars, info-mails and lectures on the subject for MHS family physicians, regarding HCV in general and our new care cascade in particular.

Finally, it is important to note that the follow-up period for the intervention group overlapped with the onset of the COVID-19 pandemic in 2020. Such a major global health event could reasonably have had an effect on compliance with cascade steps, as patients might be slightly less inclined to pursue treatment [36] and data shows that there is actually a reduction in HCV treatment rate and viremic HCV diagnosis since 2019 [37]. Nevertheless, despite these potential challenges, outcomes in the intervention group remained consistently more favorable, further supporting the effectiveness of the improved care cascade.

### Strengths and Limitations

First, the study’s design exclusively focused on new HCV diagnoses, while a sizable portion of the patients treated within these projects had prior diagnoses and either did not receive treatment or had treatment failure. In addition, a small number of patients in the RG may not have been eligible for sponsored DAAs in 2017, which may have extended their time to treatment. As various improvements were introduced gradually throughout 2019, not all patients included in the IG experienced all of the improvements implemented equally; however, logically, this would actually improve outcomes even more. SVR measurement was not defined as a follow-up goal. Additionally, the emergence of the COVID-19 pandemic early in 2020 most likely had an effect on the work of the teams, availability of tests and patient co-operation As IV drug use is rarely noted as a diagnosis, this information was not available for correlation.

This study also has several strengths. First, we were able to address several types of known barriers to treatment. Second, the unique structure of the local healthcare system allowed treatment to be accessible on a large scale and similar processes can be developed to address additional population health concerns. Third, this study population is representative of HCV positive patients in the Israeli HMOs.

## 5. Conclusions

In its pursuit of extending its effort to provide DAA treatment to all diagnosed HCV patients, MHS designed and implemented a novel program, streamlining the care cascade in order to optimize care. Our study shows that the program improved treatment uptake within 12 months of starting the cascade steps, compared to a similar group of patients diagnosed before the initiation of the new program. Analysis of key obstacles in treatment and tailoring specific changes promotes a successful diagnosis and treatment process for HCV positive patients.

## Figures and Tables

**Table 1:** Baseline characteristics of patients newly diagnosed with HCV antibody positive result in the intervention and reference groups.

Characteristic at Ab+ Result		Reference Group (n = 356)	Intervention Group (n = 328)	*p*	SMD
Age at Ab+ Test (median [IQR]), y		46.00 [38.00, 55.00]	48.00 [39.00, 57.25]	0.144	0.126
Sex. (%)	Females	146 (41.0)	127 (38.7)	0.594	0.047
	Males	210 (59.0)	201 (61.3)		
Smoking status (%)	Ever	157 (44.1)	125 (38.1)	0.035	0.224
	Never	191 (53.7)	182 (55.5)		
	Unknown	1 (0.3)	2 (0.6)		
SES (%)	Low	110 (30.9)	101 (30.8)	0.817	0.074
	Medium	171 (48.0)	165 (50.3)		
	High	71 (19.9)	60 (18.3)		
District (%)	Center	195 (54.8)	171 (52.1)	0.742	0.059
	North	82 (23.0)	83 (25.3)		
	South	79 (22.2)	74 (22.6)		
Birth country (%)	Former USSR	91 (25.6)	93 (28.4)	0.637	0.073
	Israel	244 (68.5)	219 (66.8)		
	Other	21 (5.9)	16 (4.9)		
Diabetes (%)		32 (9.0)	29 (8.8)	1.000	0.005
CKD (%)		29 (8.1)	24 (7.3)	0.793	0.031
COPD (%)		7 (2.0)	6 (1.8)	1.000	0.010
Hypertension (%)		57 (16.0)	57 (17.4)	0.707	0.037
Osteoporosis (%)		15 (4.2)	9 (2.7)	0.403	0.080
CVD (%)		2 (0.6)	3 (0.9)	0.927	0.041
HF (%)		1 (0.3)	1 (0.3)	1.000	0.004
Hepatitis B (%)		11 (3.1)	9 (2.7)	0.967	0.021
HIV (%)		7 (2.0)	2 (0.6)	0.223	0.121
HPV (%)		0 (0.0)	3 (0.9)	0.219	0.136
Chlamydia (%)		1 (0.3)	1 (0.3)	1.000	0.004
Syphilis (%)		3 (0.8)	3 (0.9)	1.000	0.008
Gonorrhea (%)		2 (0.6)	1 (0.3)	1.000	0.039
Genital herpes (%)		12 (3.4)	8 (2.4)	0.620	0.056

SES = socioeconomic status, CKD = chronic kidney disease, HF = heart failure, CVD = cardiovascular diseases, HIV = human immunodeficiency virus, HPV = human papilloma virus.

## Data Availability

The original contributions presented in this study are included in the article. Further inquiries can be directed to the corresponding author.

## Abbreviations

CBC—complete blood count, LFTs—Liver function tests, HCV—hepatitis C virus, PCR—polymerase chain reaction, DAA—direct-acting agent, MHS—Maccabi Healthcare Services, EMR—electronic medical record, SVR—sustained virological response
